# Scale-Up of Voluntary Medical Male Circumcision Services for HIV Prevention — 12 Countries in Southern and Eastern Africa, 2013–2016

**DOI:** 10.15585/mmwr.mm6647a2

**Published:** 2017-12-01

**Authors:** Jonas Z. Hines, Onkemetse Conrad Ntsuape, Kananga Malaba, Tiruneh Zegeye, Kennedy Serrem, Elijah Odoyo-June, Rose Kolola Nyirenda, Wezi Msungama, Kondwani Nkanaunena, Jotamo Come, Marcos Canda, Herminio Nhaguiombe, Ella K. Shihepo, Brigitte L.T. Zemburuka, Gram Mutandi, Emmanuel Yoboka, André H. Mbayiha, Hilda Maringa, Alfred Bere, J. Joseph Lawrence, Gissenge J.I. Lija, Daimon Simbeye, Kokuhumbya Kazaura, Ramadhani S. Mwiru, Stella Alamo Talisuna, Joseph Lubwama, Geoffrey Kabuye, James Exnobert Zulu, Omega Chituwo, Maybin Mumba, Sinokuthemba Xaba, John Mandisarisa, Brittney N. Baack, Lawrence Hinkle, Jonathan M. Grund, Stephanie M. Davis,, Carlos Toledo

**Affiliations:** ^1^Division of Global HIV and Tuberculosis, CDC; ^2^Ministry of Health and Wellness, Botswana; ^3^Division of Global HIV and Tuberculosis, CDC Botswana; ^4^Division of Global HIV and Tuberculosis, CDC Ethiopia; ^5^National AIDS and STIs Control Programme, Kenya; ^6^Division of Global HIV and Tuberculosis, CDC Kenya; ^7^Ministry of Health, Malawi; ^8^Division of Global HIV and Tuberculosis, CDC Malawi; ^9^Ministry of Health, Mozambique; ^10^Division of Global HIV and Tuberculosis, CDC Mozambique; ^11^Ministry of Health and Social Services, Namibia; ^12^Division of Global HIV and Tuberculosis, CDC Namibia; ^13^Division of Global HIV and Tuberculosis, CDC Rwanda; ^14^Ministry of Health, Rwanda; ^15^Division of Global HIV and Tuberculosis, CDC South Africa; ^16^National AIDS Control Program, Ministry of Health, Community Development, Gender, Elderly and Children, Tanzania; ^17^Division of Global HIV and Tuberculosis, CDC Tanzania; ^18^Division of Global HIV and Tuberculosis, CDC Uganda; ^19^Ministry of Health, Zambia; ^20^Division of Global HIV and Tuberculosis, CDC Zambia; ^21^Ministry of Health and Child Care, Zimbabwe; ^22^Division of Global HIV and Tuberculosis, CDC Zimbabwe.

Countries in Southern and Eastern Africa have the highest prevalence of human immunodeficiency virus (HIV) infection in the world; in 2015, 52% (approximately 19 million) of all persons living with HIV infection resided in these two regions.* Voluntary medical male circumcision (VMMC) reduces the risk for heterosexually acquired HIV infection among males by approximately 60% (*1*). As such, it is an essential component of the Joint United Nations Programme on HIV/AIDS (UNAIDS) strategy for ending acquired immunodeficiency syndrome (AIDS) by 2030 (*2*). Substantial progress toward achieving VMMC targets has been made in the 10 years since the World Health Organization (WHO) and UNAIDS recommended scale-up of VMMC for HIV prevention in 14 Southern and Eastern African countries with generalized HIV epidemics and low male circumcision prevalence (*3*).^†^ This has been enabled in part by nearly $2 billion in cumulative funding through the President’s Emergency Plan for AIDS Relief (PEPFAR), administered through multiple U.S. governmental agencies, including CDC, which has supported nearly half of all PEPFAR-supported VMMCs to date. Approximately 14.5 million VMMCs were performed globally during 2008–2016, which represented 70% of the original target of 20.8 million VMMCs in males aged 15–49 years through 2016 (*4*). Despite falling short of the target, these VMMCs are projected to avert 500,000 HIV infections by the end of 2030 (*4*). However, UNAIDS has estimated an additional 27 million VMMCs need to be performed by 2021 to meet the Fast Track targets (*2*). This report updates a previous report covering the period 2010–2012, when VMMC implementing partners supported by CDC performed approximately 1 million VMMCs in nine countries (*5*). During 2013–2016, these implementing partners performed nearly 5 million VMMCs in 12 countries. Meeting the global target will require redoubling current efforts and introducing novel strategies that increase demand among subgroups of males who have historically been reluctant to undergo VMMC.

CDC supports national ministries of health to provide VMMC services for HIV prevention in 12 priority countries: Botswana, Ethiopia, Kenya, Malawi, Mozambique, Namibia, Rwanda, South Africa, Tanzania, Uganda, Zambia, and Zimbabwe.^§^ The VMMC service package includes male circumcision, offer of HIV testing services and linkage to care and treatment for men testing HIV positive, HIV risk reduction education, condom provision, and screening and treatment or referral for sexually transmitted infections (*3*). Circumcisions are performed under local anesthesia by trained clinicians (clinical officers and nurses in most countries). All VMMC clients provide informed consent; consenting for minors adheres to national standards.

CDC-supported VMMC programs reported program data on key indicators. Data were reported in accordance with the fiscal year October 1–September 30. Data were drawn from site-level VMMC client registers, collected by VMMC implementing partners, and reported to PEPFAR and CDC. The primary indicator was the total number of VMMCs performed; disaggregated indicators included VMMC method (conventional surgical circumcision or device-based circumcision), client age group, HIV test results among VMMC clients tested at VMMC sites, and attendance at postoperative follow-up visits within 14 days.

During 2013–2014, client age was reported as <15 or ≥15 years; during 2015–2016, age was categorized as <15 years, 15–29 years, and ≥30 years. HIV prevalence was calculated by dividing the number of males that tested positive for HIV infection by the number undergoing HIV testing services at VMMC sites. In this report, disaggregated indicators were excluded from multi-country analyses if the sum of values in the disaggregated indicator was <85% or >100% of the total number of VMMCs reported for a given year.

During 2013–2016, CDC supported 4,859,948 VMMCs in 12 Southern and Eastern African countries (Table 1). The annual number of VMMCs increased during 2013–2015. In 2016, 181,737 (13.4%) fewer VMMCs were performed than in 2015. In multi-country analyses, the proportion of VMMC clients aged <15 years increased each year during 2013–2016, from 31.7% in 2013 to 47.6% in 2016 (Table 2). Conversely, the proportion of VMMC clients aged 15–29 years declined from 48.4% in 2015 to 45.6% in 2016. During 2013–2016, circumcision devices were used in 42,520 (1.1%) of the VMMCs.

**TABLE 1:** VMMCs provided in CDC-supported VMMC programs — 12 Southern and Eastern African countries, 2013–2016

Country	Fiscal year*				Total
	2013	2014	2015	2016	
Botswana	11,855	12,745	7,320	23,977	**55,897**
Ethiopia	14,037	10,439	9,861	10,655	**44,992**
Kenya	144,943	154,776	147,998	176,056	**623,773**
Malawi	18,398	18,889	18,910	19,180	**75,377**
Mozambique	121,369	141,113	159,299	184,488	**606,269**
Namibia	0	685	7,132	10,194	**18,011**
Rwanda	0	21,475	25,000	8,809	**55,284**
South Africa	139,174	185,193	193,311	149,081	**666,759**
Tanzania	159,230	278,948	341,544	181,199	**960,921**
Uganda	272,182	329,059	251,815	225,597	**1,078,653**
Zambia	96,183	154,941	147,962	126,765	**525,851**
Zimbabwe	6,171	39,840	44,868	57,282	**148,161**
**Yearly total**	**983,542**	**1,348,103**	**1,355,020**	**1,173,283**	**4,859,948**
**Cumulative total**	**983,542**	**2,331,645**	**3,686,665**	**4,859,948**	**—**

**Abbreviation:** VMMC = voluntary medical male circumcision.

* October 1–September 30.

**TABLE 2:** Disaggregated indicators for CDC-supported VMMC programs — 12 Southern and Eastern African countries, 2013–2016

Country	Fiscal year*	No. of CDC-supported VMMCs performed	No. of clients aged <15 yrs (%)	No. of clients aged 15–29 yrs (%)	No. of clients aged ≥30 yrs (%)	No. of VMMCs performed using devices (%)^†^	No. of VMMC clients receiving HIV testing services (%)^§^	No. of clients testing HIV positive (%)^¶^	No. of clients with postoperative follow-up within 14 days of VMMC (%)
Botswana	2013	11,855	4,432 (37.4)	NR (—)**	NR (—)**	807 (6.8)	11,855 (100.0)	23 (0.2)	9,880 (83.3)
	2014	12,745	8,765 (68.8)	NR (—)**	NR (—)**	64 (0.5)	12,711 (99.7)	136 (1.1)	4,572 (35.9)**
	2015	7,320	4,759 (65.0)	2,040 (27.9)	521 (7.1)	1,896 (25.9)	5,368 (73.3)	134 (2.5)	4,619 (63.1)
	2016	23,977	4,249 (17.7)**	3,660 (15.3)**	1,414 (5.9)**	2,715 (11.3)**	6,216 (25.9)**	271 (4.4)**	5,562 (23.2)**
	Total	55,897	22,205 (39.7)	5,700 (10.2)	1,935 (3.5)	5,482 (9.8)	36,150 (64.7)	564 (1.6)	24,633 (44.1)
Ethiopia	2013	14,037	56 (0.4)	11,572 (82.4)	2,409 (17.2)	0 (0.0)	13,268 (94.5)	37 (0.3)	13,905 (99.1)
	2014	10,439	1,671 (16.0)	6,880 (65.9)	1,888 (18.1)	0 (0.0)	5,802 (55.6)	4 (0.1)	10,402 (99.6)
	2015	9,861	608 (6.2)	7,339 (74.4)	1,914 (19.4)	0 (0.0)	8,081 (81.9)	9 (0.1)	9,861 (100.0)
	2016	10,655	3,194 (30.0)	6,143 (57.7)	1,318 (12.4)	0 (0.0)	4,664 (43.8)	5 (0.1)	10,597 (99.5)
	Total	44,992	5,529 (12.3)	31,934 (71.0)	7,529 (16.7)	0 (0.0)	31,815 (70.7)	55 (0.2)	44,765 (99.5)
Kenya	2013	144,943	52,643 (36.3)	NR (—)**	NR (—)**	512 (0.4)	112,657 (77.7)	1,360 (1.2)	45,300 (31.3)**
	2014	154,776	87,066 (56.3)	NR (—)**	NR (—)**	302 (0.2)	129,530 (83.7)	1,380 (1.1)	66,634 (43.1)
	2015	147,998	94,634 (63.9)	48,735 (32.9)	4,544 (3.1)	448 (0.3)	133,584 (90.3)	1,797 (1.3)	89,724 (60.6)
	2016	176,056	123,006 (69.9)	49,075 (27.9)	3,976 (2.3)	2,201 (1.3)	145,931 (82.9)	575 (0.4)	116,933 (66.4)
	Total	623,773	357,349 (57.3)	97,810 (15.7)	8,520 (1.4)	3,463 (0.6)	521,702 (83.6)	5,112 (1.0)	318,591 (51.1)
Malawi	2013	18,398	4,749 (25.8)	NR (—)**	NR (—)**	0 (0.0)	18,354 (99.8)	262 (1.4)	13,287 (72.2)
	2014	18,889	8,594 (45.5)	NR (—)**	NR (—)**	299 (1.6)	18,867 (99.9)	132 (0.7)	15,099 (79.9)
	2015	18,910	6,928 (36.6)	10,033 (53.1)	1,949 (10.3)	2,949 (15.6)	18,871 (99.8)	427 (2.3)	11,309 (59.8)
	2016	19,180	9,127 (47.6)	9,022 (47.0)	1,031 (5.4)	0 (0.0)	19,022 (99.2)	125 (0.7)	14,956 (78.0)
	Total	75,377	29,398 (39.0)	19,055 (25.3)	2,980 (4.0)	3,248 (4.3)	75,114 (99.7)	946 (1.3)	54,651 (72.5)
Mozambique	2013	121,369	62,136 (51.2)	NR (—)**	NR (—)**	0 (0.0)	123,909 (102.1)**	2,944 (2.4)**	NR (—)**
	2014	141,113	75,469 (53.5)	NR (—)**	NR (—)**	0 (0.0)	143,055 (101.4)**	1,475 (1.0)**	98,458 (69.8)
	2015	159,299	78,863 (49.5)	72,405 (45.5)	8,031 (5.0)	0 (0.0)	156,308 (98.1)	1,844 (1.2)	110,111 (69.1)
	2016	184,488	78,117 (42.3)	95,033 (51.5)	11,338 (6.1)	0 (0.0)	172,814 (93.7)	2,473 (1.4)	133,781 (72.5)**
	Total	606,269	294,585 (48.6)	167,438 (27.6)	19,369 (3.2)	0 (0.0)	596,086 (98.3)	8,736 (1.5)	342,350 (70.6)
Namibia	2013	0	NA	NA	NA	NA	NA	NA	NA
	2014	685	72 (10.5)	597 (87.2)	16 (2.3)	0 (0.0)	685 (100.0)	6 (0.9)	562 (82.0)
	2015	7,132	15 (0.2)	5,706 (80.0)	1,411 (19.8)	0 (0.0)	6,283 (88.1)	211 (3.4)	7,132 (100.0)
	2016	10,194	1 (0.0)	8,319 (81.6)	1,874 (18.4)	0 (0.0)	8,686 (85.2)	183 (2.1)	10,157 (99.6)
	Total	18,011	88 (0.5)	14,622 (81.2)	3,301 (18.3)	0 (0.0)	15,654 (86.9)	400 (2.6)	17,851 (99.1)
Rwanda	2013	0	NA	NA	NA	NA	NA	NA	NA
	2014	21,475	NR (—)**	NR (—)**	NR (—)**	0 (0.0)	17,777 (82.8)	10 (0.1)	NR (—)**
	2015	25,000	4,693 (18.8)	17,050 (68.2)	3,227 (12.9)	194 (0.8)	24,970 (99.9)	15 (0.1)	16,647 (66.6)**
	2016	8,809	593 (6.7)	7,255 (82.4)	961 (10.9)	1,336 (15.2)	8,809 (100.0)	9 (0.1)	7,454 (84.6)**
	Total	55,284	5,286 (9.6)	24,305 (44.0)	4,188 (7.6)	1,530 (3.7)	51,556 (93.3)	34 (0.1)	24,101 (71.3)
South Africa	2013	139,174	29,889 (21.5)	NR (—)**	NR (—)**	0 (0.0)	142,390 (102.3)**	4,048 (2.8)**	66,667 (47.9)
	2014	185,193	68,231 (36.8)	NR (—)**	NR (—)**	56 (0.0)	194,746 (105.2)**	4,724 (2.4)**	93,939 (50.7)
	2015	193,311	84,239 (43.6)	NR (—)**	NR (—)**	976 (0.5)	187,859 (97.2)	5,702 (3.0)	93,047 (48.1)
	2016	149,081	69,266 (46.5)	NR (—)**	NR (—)**	3,903 (2.6)	150,211 (100.8)**	6,072 (4.0)**	102,021 (68.4)
	Total	666,759	251,625 (37.7)	NR (—)	NR (—)	4,935 (0.7)	675,206 (101.3)	20,546 (3.0)	355,674 (53.3)
Tanzania	2013	159,230	64,173 (40.3)	NR (—)**	NR (—)**	0 (0.0)	NR (—)**	NR (—)**	NR (—)**
	2014	278,948	113,731 (40.8)	NR (—)**	NR (—)**	0 (0.0)	213,239 (76.4)	1,029 (0.5)	NR (—)**
	2015	341,544	142,740 (41.8)	172,594 (50.5)	26,210 (7.7)	0 (0.0)	335,105 (98.1)	926 (0.3)	312,691 (91.6)
	2016	181,199	88,607 (48.9)	79,239 (43.7)	13,353 (7.4)	0 (0.0)	180,845 (99.8)	458 (0.3)	150,605 (83.1)
	Total	960,921	409,251 (42.6)	251,833 (26.2)	39,563 (4.1)	0 (0.0)	729,189 (75.9)	2,413 (0.3)	463,296 (88.6)
Uganda	2013	272,182	54,608 (20.1)	NR (—)**	NR (—)**	NR (—)**	237,830 (87.4)	NR (—)**	NR (—)**
	2014	329,059	112,555 (34.2)	NR (—)**	NR (—)**	NR (—)**	298,060 (90.6)	NR (—)**	NR (—)**
	2015	251,815	0 (0.0)**	0 (0.0)**	466 (0.2)**	990 (0.4)**	112,465 (44.7)**	920 (0.8)**	76,432 (30.4)**
	2016	225,597	29,841 (13.2)**	35,560 (15.8)**	10,004 (4.4)**	4,168 (1.8)	215,240 (95.4)	1,144 (0.5)	173,829 (77.1)**
	Total	1,078,653	197,004 (18.3)	35,560 (3.3)	10,470 (1.0)	5,158 (0.5)	863,595 (80.1)	2,064 (0.2)	250,261 (23.2)
Zambia	2013	96,183	37,310 (38.8)	NR (—)**	NR (—)**	NR (—)**	71,407 (74.2)	491 (0.7)	77,350 (80.4)
	2014	154,941	65,481 (42.3)	NR (—)**	NR (—)**	0 (0.0)	116,881 (75.4)	1,742 (1.5)	130,360 (84.1)**
	2015	147,962	52,716 (35.6)	82,197 (55.6)	12,701 (8.6)	4,533 (3.1)	125,137 (84.6)	2,429 (1.9)	134,762 (91.1)
	2016	126,765	42,780 (33.7)	72,290 (57.0)	11,611 (9.2)	691 (0.5)	110,823 (87.4)	1,334 (1.2)	118,628 (93.6)
	Total	525,851	198,287 (37.7)	154,487 (29.4)	24,312 (4.6)	5,224 (1.0)	424,248 (80.7)	5,996 (1.4)	461,100 (87.7)
Zimbabwe	2013	6,171	2,019 (32.7)	NR (—)**	NR (—)**	0 (0.0)	6,174 (100.0)	1 (<0.1)	NR (—)**
	2014	39,840	14,827 (37.2)	NR (—)**	NR (—)**	1,085 (2.7)	39,837 (100.0)	135 (0.3)	36,566 (91.8)
	2015	44,868	19,619 (43.7)	22,453 (50.0)	2,796 (6.2)	3,452 (7.7)	44,714 (99.7)	230 (0.5)	43,180 (96.2)
	2016	57,282	24,784 (43.3)	27,065 (47.2)	5,433 (9.5)	12,648 (22.1)	57,136 (99.7)	726 (1.3)	54,772 (95.6)
	Total	148,161	61,249 (41.3)	49,518 (33.4)	8,229 (5.6)	17,185 (11.6)	147,861 (99.8)	1,092 (0.7)	134,518 (94.7)
All countries	2013	983,542	312,015 (31.7)	NA	NA	1,319 (0.1)	737,844 (75.0)	9,166 (1.2)	226,389 (23.0)
	2014	1,348,103	556,462 (41.3)	NA	NA	1,806 (0.1)	1,191,190 (88.4)	10,773 (0.9)	456,592 (33.9)
	2015	1,355,020	489,814 (44.4)	537,722 (48.7)	63,770 (5.8)	15,438 (1.1)	1,158,745 (85.5)	14,644 (1.3)	909,515 (67.1)
	2016	1,173,283	473,565 (47.0)	461,923 (45.8)	62,313 (6.2)	27,662 (2.4)	1,080,397 (92.1)	13,375 (1.2)	899,295 (76.6)
	Total	4,859,948	1,831,856 (41.2)	999,645 (47.3)	126,083 (6.0)	46,225 (1.0)	4,168,176 (85.8)	47,958 (1.2)	2,491,791 (51.3)
Multi-country analyses**	**2013**	**983,542**	**312,015 (31.7)**	**NA**	**NA**	**1,319 (0.2)**	**471,545 (83.6)**	**2,174 (0.9)**	**181,089 (64.8)**
	**2014**	**1,348,103**	**556,462 (41.9)**	**NA**	**NA**	**1,806 (0.2)**	**853,389 (83.5)**	**4,574 (0.8)**	**321,660 (58.4)**
	**2015**	**1,355,020**	**489,814 (44.4)**	**440,552 (48.4)**	**63,304 (7.0)**	**14,448 (1.3)**	**1,046,280 (94.8)**	**13,724 (1.3)**	**816,436 (75.7)**
	**2016**	**1,173,283**	**439,475 (47.6)**	**353,441 (45.6)**	**50,895 (6.6)**	**24,947 (2.6)**	**923,970 (92.4)**	**7,032 (0.8)**	**578,669 (79.2)**
	**Total**	**4,859,948**	**1,797,766 (41.5)**	**793,993 (47.1)**	**114,199 (6.8)**	**42,520 (1.1)**	**3,295,184 (89.3)**	**27,504 (1.0)**	**1,897,854 (71.9)**

**Abbreviations:** NA = not applicable; NR = not reported; VMMC = voluntary medical male circumcision.

* October 1–September 30.

^†^ Circumcision devices prequalified by the World Health Organization include the PrePex and ShangRing. However, PrePex was the predominant device in use in these 12 countries during 2013–2016.

^§^ HIV testing services exceeded 100% for certain countries that reported persons tested for HIV at VMMC clinics who did not undergo male circumcision.

^¶^ HIV prevalence was calculated by dividing the number of males that tested HIV positive by the number undergoing HIV testing services at VMMC sites.

** Excluded from multi-country analyses because the sum of values in the disaggregated indicator was <85% or >100% of the total number of VMMCs reported for the given year.

Data from multi-country analyses indicated that, during 2013–2016, 89.3% of VMMC clients participated in HIV testing services, and among those tested, the percentage of clients who tested positive ranged from 0.8% to 1.3% (at the country level, the percentage testing positive ranged from <0.1% to 4.4%) (Table 2). All VMMC clients were advised to return for a postoperative assessment; overall, 71.9% returned to the circumcising site within 14 days of surgery.

## Discussion

During 2013–2016, nearly 5 million adolescent and adult males were medically circumcised by CDC-supported VMMC programs in 12 countries in Southern and Eastern Africa. Considering that a decade ago, male circumcision was not a social norm in many of these countries, and the human and structural resources for this surgical intervention were minimal before scale-up, this represents a substantial accomplishment. In addition, many of the men who sought VMMC would not have otherwise had contact with the medical system in the absence of significant injury or illness.

However, the number of VMMCs declined in 2016, and several large-volume programs also performed fewer VMMCs in 2015. Multiple factors likely contributed to this decline, including 1) slowing of service delivery in several countries following recognition of tetanus as a rare but severe complication of VMMC, because many males in Southern and Eastern Africa were never fully immunized (*6*)^¶^; 2) retraining providers in dorsal slit circumcision technique in some countries upon identification that the forceps-guided technique posed elevated risk for injury to the immature penis (*7*); 3) prioritization of VMMC service delivery to geographic regions with the highest HIV prevalence for greater impact; and 4) possibly declining demand because many early adopters had already been circumcised.

Multiple countries increased the proportion of males aged 15–29 years who were provided VMMC in 2016, when PEPFAR began emphasizing prioritizing VMMC in this age group to most immediately achieve the HIV preventive benefit of VMMC (*8*); however, the overall percentage of males aged 15–29 years who were circumcised declined in 2016. CDC continues to work with partners to identify and implement innovative approaches to increase VMMC demand among these men (*9*). The large proportion of VMMC clients aged <15 years also likely accounts for the lower HIV prevalence observed among VMMC clients compared with national estimates,** because many of those aged <15 years likely had not yet had sexual intercourse, the primary mode of HIV transmission in this setting.

The findings in this report are subject to at least four limitations. First, the findings reflect results from CDC-supported VMMC programs rather than national, PEPFAR, or global totals. Data entry errors and reporting variations are possible, and data were incomplete for some countries in some years. Second, during 2013–2014, the disaggregated age group indicator definition prevented reporting on males aged 15–29 years. Third, use of HIV testing services did not include clients with indeterminate results or those who might have been tested elsewhere recently, possibly affecting the HIV prevalence estimate among VMMC clients. Finally, follow-up within 14 days was likely underestimated because reported data might not include males who sought care at another health care site different from the one where they underwent circumcision.

VMMC is an evidence-based, one-time intervention that confers lifelong partial protection against HIV infection for males. In addition, its benefits carry over to females by lowering the prevalence of HIV (and several other sexually transmitted infections) among potential sex partners (*10*). To date, significant progress has been made by countries with VMMC programs. However, many more VMMCs need to be performed to reach the ambitious UNAIDS target by 2021. Enhancing VMMC service delivery will involve simultaneous focusing on supply-side and demand-side factors. On the supply side, VMMC programs are 1) offering service delivery on days and times that best match clients’ needs, including evening and weekend hours; 2) using mobile outreach service delivery to overcome geographic barriers; 3) ensuring safe service delivery through quality improvement and assurance activities and rigorous adverse event monitoring (*6*); 4) where possible, layering VMMC service delivery with other health care services such as preexposure prophylaxis, HIV care and treatment, and general medical care; and 5) incorporating medical innovations (e.g., new circumcision devices) that might enhance acceptability of VMMC for some males.

To increase demand for VMMC, programs are 1) evolving messaging from generating general awareness to addressing specific concerns of persons who have been hesitant to undergo VMMC; 2) linking VMMC with prevention activities for women (e.g., perinatal HIV testing services and HIV prevention programs that target adolescent girls and young women [i.e., the DREAMS program^††^]); 3) engaging community stakeholders, such as traditional and religious leaders, celebrities, and satisfied VMMC clients, to become VMMC champions; 4) compensating clients for the opportunity cost of undergoing VMMC; and 5) ensuring VMMC services are available to men regardless of HIV status, through voluntarism of HIV testing services. Going forward, country programs at or nearing targets should begin planning for VMMC program sustainability, including VMMC training and program staffing operated by ministries of health, regional or national government contributions to VMMC financing, and establishing a framework to maintain high male circumcision coverage by continuing a VMMC program for adolescents males aged 10–14 years and/or introducing routine early infant male circumcision. Reaching and maintaining high male circumcision coverage in countries with high prevalence of HIV infection remains a critical component of achieving an AIDS-free generation.

**Summary**

**What is already known about this topic?**

Voluntary medical male circumcision (VMMC) has been recognized by the World Health Organization and Joint United Nations Programme on HIV/AIDS as an effective human immunodeficiency virus (HIV) infection prevention intervention in settings with a generalized HIV epidemic and low male circumcision prevalence. During 2010–2012, CDC (through the U.S. President’s Emergency Plan for AIDS Relief) supported 1,020,424 VMMCs in nine countries in Southern and Eastern Africa.

**What is added by this report?**

During 2013–2016, CDC-supported implementation partners performed 4,859,948 VMMCs in 12 countries in Southern and Eastern Africa, a substantial increase from 2010–2012.

**What are the implications for public health practice?**

Although millions of males have been medically circumcised in CDC-supported programs, many more VMMCs need to be performed to reach global targets. This will require redoubling current efforts and introducing novel strategies that increase demand among subgroups of males who have historically been reluctant to undergo VMMC.

## Footnotes

* http://aidsinfo.unaids.org/.

† In 2015, the prevalence of HIV infection among all persons (male and female) aged 15–49 years in the 14 priority VMMC countries was as follows: Botswana (22.2%), Ethiopia (not available), Kenya (5.9%), Lesotho (22.7%), Malawi (9.1%), Mozambique (10.5%), Namibia (13.3%), Rwanda (2.9%), South Africa (19.2%), Swaziland (28.8%), Tanzania (4.7%), Uganda (7.1%), Zambia (12.9%), and Zimbabwe (14.7%).

§ CDC support includes hiring of clinical staff members to provide VMMCs, conducting training and quality assurance assessments, providing technical assistance, and procurement of VMMC supplies, medications, and instruments.

¶
http://apps.who.int/iris/bitstream/10665/250146/1/WHO-HIV-2016.19-eng.pdf .

** In 2015, the prevalence of HIV infection among males aged 15–24 years in the 14 priority VMMC countries was as follows: Botswana (3.9%), Ethiopia (not available), Kenya (2.3%), Lesotho (5.1%), Malawi (1.8%), Mozambique (2.3%), Namibia (2.4%), Rwanda (0.8%), South Africa (4.0%), Swaziland (7.3%), Tanzania (1.0%), Uganda (1.9%), Zambia (3.1%), and Zimbabwe (3.8%). The prevalence among males aged 15–49 years was as follows: Botswana (17.8%), Ethiopia (not available), Kenya (4.8%), Lesotho (18.9%), Malawi (7.1%), Mozambique (8.7%), Namibia (10.7%), Rwanda (2.3%), South Africa (14.9%), Swaziland (23.2%), Tanzania (3.7%), Uganda (5.9%), Zambia (10.9%), and Zimbabwe (12.1%).

††
https://www.pepfar.gov/partnerships/ppp/dreams/ .
